# Artificial intelligence in screening and diagnosis of surgical diseases: A narrative review

**DOI:** 10.3934/publichealth.2024028

**Published:** 2024-04-23

**Authors:** Giuseppe Palomba, Agostino Fernicola, Marcello Della Corte, Marianna Capuano, Giovanni Domenico De Palma, Giovanni Aprea

**Affiliations:** 1 Department of Clinical Medicine and Surgery, University of Naples, “Federico II”, Sergio Pansini 5, 80131, Naples, Italy; 2 Azienda Ospedaliera Universitaria San Giovanni di Dio e Ruggi d'Aragona - OO. RR. Scuola Medica Salernitana, Salerno, Italy

**Keywords:** artificial intelligence, artificial intelligence diagnosis, artificial intelligence screening, general surgery AI diagnosis, CAD systems, CNN diagnosis

## Abstract

Artificial intelligence (AI) is playing an increasing role in several fields of medicine. It is also gaining popularity among surgeons as a valuable screening and diagnostic tool for many conditions such as benign and malignant colorectal, gastric, thyroid, parathyroid, and breast disorders. In the literature, there is no review that groups together the various application domains of AI when it comes to the screening and diagnosis of main surgical diseases. The aim of this review is to describe the use of AI in these settings. We performed a literature review by searching PubMed, Web of Science, Scopus, and Embase for all studies investigating the role of AI in the surgical setting, published between January 01, 2000, and June 30, 2023. Our focus was on randomized controlled trials (RCTs), meta-analysis, systematic reviews, and observational studies, dealing with large cohorts of patients. We then gathered further relevant studies from the reference list of the selected publications. Based on the studies reviewed, it emerges that AI could strongly enhance the screening efficiency, clinical ability, and diagnostic accuracy for several surgical conditions. Some of the future advantages of this technology include implementing, speeding up, and improving the automaticity with which AI recognizes, differentiates, and classifies the various conditions.

## 1. Introduction

Artificial intelligence (AI) is a discipline that studies models and the development of algorithms so that machines can acquire the ability to self-learn and achieve human-like performance in complex tasks [1],[2]. These algorithms are based on the analysis of large amounts of data in a limited time span to achieve learning, problem-solving, and decision-making in complex scenarios [3]. AI represents, therefore, a tool with the potential to revolutionize the practice of medicine in many fields, including surgery.

Today's medicine has reached a complex and at times overwhelming level of data production. Analyzing and retaining such a plethora of data is not always feasible in a limited time span, and it may require skills that could prove difficult to be retained by a single expert [4]. Therefore, machines with deep learning capabilities can be used as an invaluable tool for *observing* data and learning from them. The aim is to support doctors in their daily clinical practices [5], reducing diagnostic and screening errors, which are still an important cause of foreseeable and preventable damage to patients.

Indeed, studies are being published on the role already played by AI-based decision-making algorithms in supporting doctors at the point of care [6],[7]. Furthermore, it is foreseeable that predictive analytics could generate personalized, patient-centered decision-making, improving care and diagnosis [8].

The World Health Organization (WHO) has suggested various types of screening procedures, depending on the type of cancer being examined (e.g., mammography for the breast, pap smear for the cervix). However, they still have limitations [9] such as the operator-dependent variability in the reporting of an exam and the long analysis time of some data-rich techniques, such as MRI. To overcome these objective difficulties, doctors have been supported by tools in their clinical practice. These tools are known as computer-aided detection and diagnosis (CAD) systems. Through diagrams and mathematical models, CAD systems have the task of analyzing data [10]. This feature removes the difficulties due to inter- and intra-observer variability and reduces the effort required to perform the analysis [11].

Despite its potential, the development of medical AI systems as diagnostic aids represents an untapped opportunity, especially in surgery [2],[8],[12]–[14]. In fact, there is still some mistrust of AI among surgeons, which makes its adoption in decision-making less likely [2],[14].

In the literature, there are only a few studies on the applications of AI in the screening and diagnosis of surgical conditions. However, the subject of AI in general surgery is gaining an increased interest and relevance as a potential diagnostic and therapeutic tool [10]. Historically, surgery has been one of the branches most inclined to innovation and adoption of new technologies able to offer advantages in terms of performance [14]. To our knowledge, this is the first review that analyzes the application of AI in the diagnosis and screening of some of the main diseases with surgical interest; it aims to provide an in-depth analysis of the use and future applications of AI in fields such as gastric cancer, colorectal cancer, or thyroid cancer.

## 2. Methods

**Figure 1.**
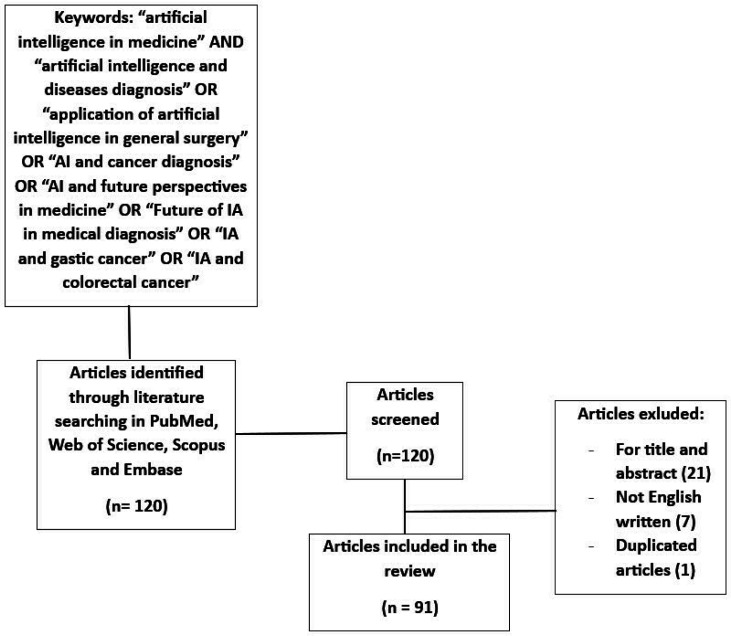
Flowchart of research.

We performed a literature review by searching PubMed, Web of Science, Scopus, and EMBASE using the following keywords: “artificial intelligence in medicine” AND “artificial intelligence and diseases diagnosis” OR “application of artificial intelligence in general surgery” OR “AI and cancer diagnosis” OR “AI and future perspectives in medicine” OR “Future of AI in medical diagnosis” OR “AI and gastric cancer” OR “AI and colorectal cancer”. Articles published between January 2000 and June 2023, addressing the use of AI as a diagnostic aid in general surgery, were analyzed (Figure 1). The search was limited to studies in the English language. Incomplete articles and those in preprint or not peer-reviewed were excluded. We focused our attention on randomized controlled trials (RCTs), meta-analyses, systematic reviews, and observational studies on cohorts of patients. The process of screening and selecting articles was based on titles, abstracts, and full-text reviews. Our focus was to evaluate the various studies addressing the association between AI and screening or diagnosis of the main diseases in the field of general surgery (colorectal, stomach, thyroid, parathyroid, breast). We also aimed to evaluate any prospects for the application of AI in the same field of interest.

From a total of 120 articles analyzed, 91 items were selected and 29 excluded, as shown in Figure 1. The articles included were either prospective or retrospective, monocentric or multicenter studies, and with a variable number of patients (hundreds to thousands).

## 3. Results

The results of our review have been organized into paragraphs, divided into fields of application of AI in colorectal, stomach, thyroid, parathyroid, and breast diseases. The Tables 1, 2, 3 and 4 show observational studies divided by topic.

### 3.1. AI in colorectal cancer

Screening, diagnosis, and treatment of colorectal cancer (CRC) may represent an important field of application of AI [15]–[17]. Colorectal cancer is the third most frequent cancer in the world; however, through screening and early diagnosis programs, its mortality rate is being reduced [18].

A few studies have evaluated the integration of AI algorithms with gene expression in CRC patients. Hu et al. showed the ability of AI to detect an eventual relapse after surgery [19]. The authors compared the performance of three neural networks: *S-Kohonen*, *Back-propagation*, and *Support Vector Machine* (SVM) [19]. The AI tool analyzed the presence of genes predictive of malignancy. The genes were extrapolated from tissue samples obtained from 53 patients who had undergone surgery for CRC. *S-Kohonen* was the best at correctly recognizing the largest number of genes associated with cancer [19]. Xu et al., using the SVM system, identified 15 genes as predictors of risk of recurrence or prognosis in patients with colon cancer [20].

An interesting study, published in 2019 by Wang et al., proposed new approaches to improve the diagnosis of CRC using The Cancer Genome Atlas database and analyzing artificial neural networks. These authors have identified four diagnostic models with a predictive accuracy of 100%: Cancer/Normal, M0/M1, carcinoembryonic antigen (CEA) <5/≥5, and Clinical stage I–II/III–IV [21]. Other studies have evaluated the ability of artificial neural networks to compare the expression profiles of micro RNAs (miRNAs) in CRCs. MiRNAs could be potential drug targets [22]–[24].

Another field of application of AI could be the assessment of CRC risk in the general population [17]. Colonoscopy is the gold standard screening test for CRC. However, there is a percentage of CRCs that present a few years after a negative colonoscopy [17],[25].

Some studies have shown the role of AI during colonoscopy in the automated identification and characterization of polyps through the use of CAD [12]–[15]. The neural network used was a convolutional neural network (CNN). CNN helps to interpret complex images by simplifying the detection of premalignant lesions [17]. Wang et al. showed that a colonoscopy with CNN achieved a better identification of hyperplastic polyps and small polyps compared with a colonoscopy alone [26]. This neural network identified the region of interest (ROI) in a complex image, discerning the lesions present in the ROI as polyposis or non-polyposis [26].

Further studies have highlighted the ability of AI algorithms to detect colorectal polyps with high sensitivity and specificity [27]–[30]. Deliwala et al., in their meta-analysis of six RCTs totaling approximately 5000 patients, concluded that AI is useful in detecting small-sized adenomas [28]. Hori et al. developed an automated collection system using a deep learning algorithm, which allowed the collection of 47,000 endoscopic images taken from approximately 750 colonoscopies and the extraction of key polyp images [29]. The AI system identified 1356 images of polypoid lesions with 97% sensitivity, 97.7% specificity, and 97.3% accuracy [29]. Hassan et al. used AI to distinguish between adenomatous and non-adenomatous lesions smaller than 5 mm in the sigmoid colon and the rectum [30]. In 291 of the 295 lesions, the AI technology was able to correctly diagnose a polypoid lesion [30].

The Synergy-Net project used AlexNet with promising results [31]. This CNN was previously trained on a dataset of images of neoplastic lesions. The dataset used was the ImageNet, obtained from approximately 200 colonoscopy images, selected by surgeons and gastroenterologists [31]. From these images, AlexNet identified the large polypoid lesions. The future prospect is to use AlexNet during a colonoscopy [31].

Kudo et al. used AI to identify patients with lymph node metastases in T1 colorectal cancer [32]. AI recognized lymph node metastases in 10.2% of CRC patients (319 out of 3134), showing the potential to be used in the foreseeable future to assess the risk of lymph node metastases.

Based on this new evidence, routine colonoscopy can benefit from the help of AI-assisted models, which can be used as an “extra pair of eyes” in real time [17].

There are also interesting studies on the use of AI in identifying complete responders (CR) and non-responders (NR) to neoadjuvant chemoradiotherapy (CRT) in locally advanced rectal cancer (LARC) [33]–[36].

The role of AI in surgical procedures is still uncertain. With the development of robotic surgery, algorithms could also be developed in the future that could help the surgeon locate planes of dissection and perform a complete lymphadenectomy.

**Table 1.** Observational studies on artificial intelligence in colorectal cancer.

**Authors**	**Year**	**Design**	**Type of study**	**Sample size**
Hu et al. [19]	2015	Prospective	Single center	53
Xu et al. [20]	2016	Retroscpective	Genetic screening	1207 genes
Morris et al. [25]	2015	Retrospective	Multicenter	94,648
Wang et al. [26]	2019	Prospective	Single center	1058
Hori et al [29]	2022	Prospective	Single center	47,391 endoscopic images
Deliwala et al. [28]	2021	Prospective	Multicenter	4996
Hassan et al. [30]	2022	Prospective	Single center	162
Kudo et al. [32]	2021	Prospective	Multicenter	3134
Ferrari et al. [33]	2019	Prospective	Single center	55
Shi et al. [34]	2019	Prospective	Single center	51
Abraham et al. [35]	2021	Prospective	Single center	467
Oyaga-Iriarte et al. [36]	2019	Prospective	Single center	20

### 3.2. AI in gastric cancer

Gastric cancer is the fourth most common cancer in men and the seventh most common in women [37]. The 5-year survival for stage IA and IB cancers treated with surgery ranges from 60% to 80%. Patients with stage-III tumors who undergo surgery have a 5-year survival rate of 18% to 50%, depending on the dataset [38],[39]. Diagnosis of gastric cancer is often delayed [40]. This is due, in many cases, to an insidious onset with non-specific and latent symptoms. According to various studies, an early diagnosis of gastric cancer can improve the 5-year survival rate [41].

Early detection of cancerous lesions is crucial to prolong survival; however, its accuracy is operator-dependent. To overcome the risk of inconsistencies, AI is taking on an important role in detecting gastric lesions during gastroscopy through the automatic recognition of ROI [42]–[45].

Luo et al. trained a neural network with 1,036,496 standard white-light endoscopic images of 84,424 cases across China [42]. The sensitivity achieved by their AI was comparable to that of an experienced endoscopist and higher compared with competent and trainee endoscopists. Consequently, the authors developed the Gastrointestinal AI Diagnostic System (GRAIDS).

Sakai et al. trained a neural network to detect cancerous lesions and distinguish them from normal stomach images during gastroscopy [43]. The method used was transfer learning. The ability of AI to automatically discriminate between the two types of gastroscopic images was 87.6%.

Hirasawa et al. used an AI tool, trained with over 13,000 gastroscopic images, with the aim of recognizing both early and advanced cancer images [44]. The AI tool was tested on 71 cancer images with a diameter greater than or equal to 6 mm. In 70 of the 71 cases, AI was able to identify the neoplastic lesion, with an accuracy of 98.6%.

Wu et al. trained a neural network to recognize early gastric cancer (EGC) [45]. The neural network demonstrated an accuracy of 92.5%, a sensitivity of 94.0%, a specificity of 91.0%, a positive predictive value (PPV) of 91.3%, and a negative predictive value (NPV) of 93.8% [45]. In this case, AI was exposed to a grid model that fractionated the stomach into different windows. The AI automatically signaled blind gastric regions to the endoscopists during EGD. When the gastroscope was inserted into the stomach, the neural network model started capturing gastric images and filling them in the corresponding part of the model it was trained on, coloring the various parts (so-called ROIs).

An important application of AI is the recognition of precancerous gastric lesions. Again, the recognition of precancerous lesions, endoscopically, often depends on the experience of the endoscopist [46]. For this reason, Guimarães et al. trained a neural network that recognized chronic atrophic gastritis [47]. Being trained with 200 endoscopic images of chronic atrophic gastritis, AI reached an accuracy of 93% in recognizing these precancerous lesions, faring better than a team of expert endoscopists.

Some authors have focused on the ability of AIs to distinguish malignant from non-malignant lesions. Horiuchi et al. trained their neural network with 1492 EGCs and 1078 gastritis images [48]. AI correctly recognized 220 of the 258 images submitted sequentially to the neural network, with a sensitivity of 95.4%, specificity of 71%, and PPV of 82.3%.

Ueyama et al. trained a neural network with 5574 endoscopic images of both neoplastic and non-neoplastic lesions [49]. The overall accuracy, sensitivity, and specificity of this system were 98.7%, 98%, and 100%, respectively.

Li et al. developed a neural network called *GastricNet* to automatically identify cancerous gastric lesions [50]. The classification accuracy of the proposed picture was 100% on gastric pathological sections.

Even in computed tomography (CT), the ability to make a correct diagnosis of gastric cancer is operator-dependent [51]. Some authors developed AIs capable of identifying ROIs in CT images. Huang et al. created a neural network model to identify preoperative peritoneal metastases in advanced gastric cancer [52]. Li et al. proposed CT imaging analysis for lymph node metastases in gastric cancer [53]. Their algorithm developed in 2012 achieved 96.3% accuracy; the algorithm developed in 2015 achieved an accuracy of 76.9% [54].

Another field of study in gastric cancer is a preoperative criterion for endoscopic resection: the depth of tumor invasion. Endoscopic curative resection can be performed for intramucosal cancer and cancer with submucosal invasion <500 µm [55]. Studies used transfer learning technology to instruct AI to predict the depth of invasion [56],[57]. Zhu et al. and Nagao et al. used a pre-trained CNN with 203 and 16,557 gastric cancer images, respectively. Both AI technologies achieved greater accuracy than human endoscopists in predicting any submucosal invasion by looking at the endoscopic image [56],[57].

Another application of AI concerns the predictive ability of patient survival with gastric cancer. Jiang et al. developed an AI that ranked patients on survival prognosis, which included the survival capacity of patients undergoing adjuvant chemotherapy [58]. According to the authors, the AI's ability to predict the survival rate of patients was superior to the staging system developed by the *American Joint Committee on Cancer*. Lu et al. performed a similar study to Jiang's, demonstrating that their AI model improved the ability to predict overall survival [59].

Another application of AI, which aims to determine the patient's prognosis, is the recurrence risk assessment, particularly when it comes to the risk of developing metastases. Hensler et al. devised an AI capable of predicting lymph node metastases preoperatively [60]. Their model was superior to the diagnostic system of the *National Cancer Center* in Tokyo. Jagric et al. have devised an AI capable of identifying the risk of liver metastases in patients who have already been operated for gastric cancer [61]. Jiang et al. have developed a deep learning model to predict peritoneal recurrence of gastric cancer from preoperative CT images [62]. The number of patients enrolled in the study was 2320. The AI technology showed an ability to predict peritoneal recurrence with a steadily increased accuracy. The use of this AI improved disease-free survival. When AI classified the patient with a high risk of peritoneal recurrence, postoperative adjuvant chemotherapy markedly improved disease-free survival. Consequently, when the predicted risk of AI was low, adjuvant chemotherapy did not improve disease-free survival.

**Table 2.** Observational studies on artificial intelligence in gastric cancer.

**Authors**	**Year**	**Design**	**Type of study**	**Sample size**
Luo et al. [42]	2019	Retrospective	Multicenter	1,036,496 endoscopy images
Hirasawa et al. [44]	2018	Prospective	Single center	69
Wu et al. [45]	2019	Prospective	Single center	324
Horiuchi et al. [48]	2019	Retrospective	Single center	2570 endoscopy images
Ueyama et al. [49]	2021	Retrospective	Single center	5574 endoscopy images
Li et al. [50]	2020	Prospective	Multicenter	341
Zhu et al. [56]	2019	Retrospective	Single center	993 endoscopy images
Nagao et al. [57]	2020	Prospective	Single center	1084
Jagric et al. [61]	2010	Retrospective	Single center	213

### 3.3. AI in breast cancer

One of the main fields of application of AI is breast cancer screening and diagnosis [63]. AI has been used in the recognition of breast lesions by targeting specific ROI. After receiving a radiological or tomographic image of the breast, AI breaks it down into smaller images and automatically identifies the regions of interest. ROIs are areas automatically selected by AI where the likelihood of detecting the breast lesion is likely to be higher [63].

Approximately 97% of mammograms show no malignant breast lesions (identifiable as true negatives). Screening mammography has been shown to reduce breast cancer mortality. Given the large number of mammograms performed each year (33 million in the US alone), automating the diagnosis would reduce the workload of radiologists, improving reporting speed [64].

The task of AI is to help the radiologist in the diagnosis of breast lesions that are considered suspicious or that were not considered in the evaluation of mammography or tomography. The goal is to reduce false negatives [65]. Retrospective studies were therefore carried out to evaluate the ability of CAD systems to identify breast lesions in radiographic and tomographic images.

The initial results appear promising [66]–[69]; however, a study conducted by Lehman et al. showed that CAD systems detected a larger number of false positives [70]. Indeed, in recent years, new protocols have been expected to improve the screening intervals and the technologies used but, in turn, they can increase false positives [71].

Some risk factors for breast cancer are radiologically relevant; a particularly important one is breast density. In fact, dense breasts can hide lesions in mammograms, carrying a higher risk of causing false negatives. Hence, AI is gaining an increasingly important role in breast cancer diagnostics [72]. The ability to evaluate a breast lesion in a dense breast depends on the experience of the operator assessing the mammogram [73]. Some authors have trained AI with numerous radiological images and assessments made by expert radiologists. Mohamed et al. trained AlexNet using mammograms from 1427 women [74]. The authors demonstrated that the ability of AI to identify breast lesions in dense breasts depends on the type of mammographic projection used, showing greater precision in mediolateral oblique views.

Lehman et al. developed an AI technology called ResNet-18 using mammography images from 39,272 women [75]. Initially, the assessment by the AI was compared with that of 12 radiologists, showing a good level of agreement. Subsequently, 500 images were randomly selected and subjected to the opinion of five other radiologists; also, in this case, there was a good level of agreement. Finally, eight other radiologists reviewed 10,763 images already evaluated by the AI, with similar levels of agreement.

Dontchos et al. used a deep learning model for predicting mammographic breast density in routine clinical practice [76]. They compared mammogram assessments by academic and non-academic radiologists (94.9% and 90.7%). The result was a reduction in the percentage of mammograms rated as dense from 47% to 41%.

Kallenberg et al. developed a deep learning model to predict the risk of breast injury associated with breast density [77]. The sample included 493 women. The two inputs used were breast density segmentation and mammography facility score. Sørensen–Dice coefficient (DSC) was 63%; this means that in 63% of cases, there was a similarity between the predictive model of breast injury and reality.

Finally, some AI studies evaluated the assessment of breast density from reconstructed 3D images. In Gastounioti et al., 132 women already diagnosed with breast cancer and 528 women with no evidence of neoplastic breast lesions were enrolled in the study [78]. A software reconstructed the breasts in 3D; then, these images were subjected to pre-trained AI to associate breast cancer risk with breast density. The results were adjusted for age and body mass index. The mean age was similar for the two groups, approximately 60 years. Some images were obtained from mammograms (DM) and others from digital tomosynthesis (DBT). Volume density estimates calculated from DBT were more strongly associated with breast cancer than density derived from DM for both groups.

**Table 3.** Observational studies on artificial intelligence in breast cancer.

**Authors**	**Year**	**Design**	**Type of study**	**Sample size**
Birdwell et al. [66]	2001	Retrospective	Multicenter	110 patients
Warren Burhenne et al. [67]	2000	Retrospective	Multicenter	427 (images)
Freer et al. [68]	2001	Prospective	Single center	12,860 (images)
Destounis et al [69]	2004	Retrospective	Single center	318 (images)
Lehman et al. [70]	2015	Prospective	Multicenter	323,973 (images)
Dontchos et al. [76]	2021	Prospective	Multicenter	2174 (images)

### 3.4. AI in parathyroid and thyroid disease

In recent years, the use of AI for the screening and diagnosis of parathyroid and thyroid diseases has been increasingly used in several research programs [79]. At the time of surgery, one of the difficulties may be the identification of some anatomical structures. A typical difficulty is distinguishing parathyroid glands from perithyroidal lymph nodes [80].

Wang et al. addressed this problem by using three AI models, named *YOLO V3, Faster R-CN and Cascade* [81]. These AI models were pre-trained to recognize the parathyroids by using images from 166 videos, showing a total of 1700 parathyroids. The algorithm with the best performance in recognizing parathyroids was R-CNN. Data from 20 additional full-length videos were used as an independent external cohort for two groups of professionals: one of junior surgeons and one of senior surgeons, to compare the accuracy of parathyroid recognition by AI with the two groups. The accuracy of AI was 88.7%, which compared favorably to the independent external cohort of senior and junior surgeons, whose detection rates were 87.5% and 71.9%, respectively. Furthermore, AI recognized the parathyroid glands with an advantage of 3.83 s compared to senior surgeons, with a longer monitoring period of 62.82 s compared to junior surgeons. From these results, the ability of parathyroid recognition by AI appeared superior to that of junior surgeons and like that of experienced surgeons. In the future, such AI models could be used to speed up and simplify the recognition of parathyroids, assisting especially junior surgeons.

Another study was conducted by Seyma et al. [82]. The authors developed an AI technology able to recognize abnormal parathyroids (e.g., hypersecretory ones) during surgery and differentiate them from normal parathyroids. The parathyroids are known to fluoresce darker and heterogeneously in cases of hyperparathyroidism [83],[84]. A total of 303 patients were enrolled in the study, and 906 auto-fluorescent intraoperative parathyroid images were used to train and test the AI model. The goal of AI was to recognize and differentiate abnormal parathyroids. Area under the receiver operating characteristic curve (AUROC) and area under the precision-recall curve (AUPRC) of the model to predict normal and abnormal parathyroid glands were 0.90 and 0.93, respectively. Model recall and model accuracy were 89% each. From their results, the authors concluded that AI could be used effectively in cases of primary hyperparathyroidism by recognizing abnormal parathyroids during surgery [83],[84].

Regarding thyroid surgery, one of the difficulties of surgeons is to identify lymph node masses suspected of being positive for metastases [85]. Techniques have been developed to identify and differentiate normal from abnormal lymph nodes. In patients with papillary thyroid cancer (PTC), the presence or absence of lymph node metastases has crucial implications for both staging and treatment of the disease, although lymph nodes are often not removed during thyroidectomy.

Esce et al. developed a CNN to predict lymph node metastases starting from the histology of PTC [86]. A cohort of 174 patients was enrolled to train AI so that it could discriminate the group with lymph node metastases from the group without lymph node metastases. The neural network recognized lymph node metastases with a sensitivity of 94% and a specificity of 100%. Based on their results, the authors suggested that, in the future, AI will be usable to identify lymph node metastatic lesions from visual histopathological images.

Recently, the same authors conducted a follow-on multicenter study aimed at further assessing the ability of a CNN to predict the presence or absence of lymph node metastases in patients with PTC [87]. When CNN from one institution was tested against images from the other institution, the achieved sensitivity and specificity were 65% and 61%, respectively. The best-performing institution's combined algorithm had a sensitivity and specificity of 68% and 91%, respectively. The authors concluded that the results were comparable to the ones obtained in their previous study, confirming the future potential advantage of using AI technologies to generate algorithms able to predict the presence of lymph node metastases in patients with PTC [87].

Wang et al., in turn, developed an AI model to recognize cervical lymph node metastases [88]. In their retrospective multicenter study, preoperative CT images were used to train the AI neural network. An experienced radiologist identified an ROI for each CT scan. Subsequently, the same CT images were used to train the AI. With the help of the AI system, the radiologists' specificity was improved by 9% and 15% for R1 and 13% and 9% for R2, respectively; this means that the AI model was superior to the radiomic and clinical model in predicting the cervical lymph node metastasis (CLNM) of the PTC. Therefore, increasing the preoperative diagnostic capabilities could also improve the accuracy and precision of the surgical intervention in the future.

Ultrasonography is an essential diagnostic tool for the early identification of benign and malignant thyroid lesions [89],[90]. However, being operator-dependent, its diagnostic yield may be reduced in the hands of young or less experienced doctors. Therefore, the use of AI can assist sonographers in improving their diagnostic accuracy while reducing the time to diagnosis [91]. One of the main applications of AI-assisted ultrasonography involves the discrimination between benign and malignant thyroid nodules. Several authors have developed CAD that uses machine learning (MC) technologies with an aim to demonstrate the ability of AI to assist doctors in the diagnosis of thyroid diseases [91].

The first to carry out such a study were Hirning et al. [92]. Already in 1989, the authors enrolled 55 patients and created an automatic classifier of benign and malignant thyroid lesions, achieving an accuracy of 85%.

Tsantis et al. and Chang et al. performed the same type of study on 120 and 61 patients, reaching accuracies of 96.7% and 100%, respectively [93],[94].

Iakovidis et al. were among the first to use a fuzzy system to classify thyroid lesions by ultrasound [95]. Fuzzy systems appear to be suitable methods for examining data as they deal with uncertainty and ambiguity in data [96]. They have been successfully applied to various areas such as classification, pattern recognition, and prediction.

AI has also been used to identify benign and malignant thyroid nodules in fine needle aspiration biopsies (FNAB). Kezlarian et al. have published a systematic review on this topic [97]. The authors did not find statistically significant reliability at the point of care, deeming algorithms not yet suitable for correctly classifying benign and malignant FNABs [97].

Some authors have tried to use AI to distinguish follicular adenomas from follicular carcinomas. Currently, the diagnosis requires evaluation of the thyroid capsule. For this reason, the authors have trained AI technologies. Savala et al. used 57 cases divided between adenomas and follicular carcinomas [98]. The AI tool recognized three follicular adenomas and six follicular carcinomas.

Shapiro et al. trained three neural networks with the same goal [99]. The first used cytology features such as colloid, cytoplasm, and tissue fragments selected by expert pathologists, the second used thyroid morphology features, and the third used images of Giemsa-stained smears. The cytological algorithms correctly classified 93%, 96%, and 87% of cases, respectively.

Other authors have been developing an AI classifier that identifies benign and malignant thyroid nodules based on their ultrasound features. Chen et al. enrolled 227 participants with 256 nodules [100]. Patients' clinical data were collected and the presence of calcifications on conventional grayscale ultrasound images was retrospectively examined by an endocrinologist. Quantification of cystic components and calcifications was performed automatically by the classifier (AmCAD-UT). The calcification index (CI) was calculated after excluding the cystic component. Calcifications were found in 48.19% of malignant thyroid nodules and 10.98% (19 of 173) of benign nodules [100]. This new computer-assisted diagnosis method for evaluating ultrasound calcifications of thyroid nodules is a more sensitive and objective method. According to the authors, it may provide better sensitivity than conventional methods in the diagnosis of thyroid tumors containing microcalcifications. Choi et al. used images of 99 calcified thyroid nodules to train a neural network [101]. The neural network subsequently had to be capable of classifying benign and malignant thyroid lesions. The data examined dealt with the relationship between calcification distance, number of calcifications, and maximum intensity. The results showed a sensitivity of 83.0%, a specificity of 82.4%, and an accuracy of 82.8% [101].

**Table 4.** Observational studies on artificial intelligence in parathyroid and thyroid disease.

**Authors**	**Year**	**Design**	**Type of study**	**Sample size**
Wang et al. [81]	2022	Prospective	Single center	1700 images
Avci et al. [82]	2022	Prospective	Single center	303 images
Akbulut et al. [83]	2021	Prospective	Single center	106 images
Esce et al. [86]	2021	Prospective	Single center	174 patients
Esce et al. [87]	2023	Retrospective	Multicenter	420 patients
Wang et al. [88]	2023	Retrospective	Multicenter	671 patients
Tsantis et al. [93]	2005	Prospective	Single center	120 images
Iakovidis et al. [95]	2010	Prospective	Single center	75 patients
Savala et al. [98]	2018	Prospective	Single center	57 images
Shapiro et al. [99]	2007	Prospective	Single center	197 images
Chen et al. [100]	2011	Prospective	Single center	227 patients
Choi et al. [101]	2015	Retrospective	Single center	99 images

## 4. Limits and future prospects

AI provides advantages when applied to the diagnosis and screening processes of surgical diseases. AI systems are useful in helping surgeons to identify precancerous lesions and anatomical structures of doubtful interpretation, as well as evaluating the accuracy of a diagnostic and screening procedure. They also prove to be useful in developing precision medicine models personalized for each individual patient. All this can improve the speed and accuracy of some diagnoses.

However, there are still significant limitations to the use of AI in the clinical setting. First, it is not easy to train an AI to think like a human and use it on a large scale. The standardization of AI and its uses by governmental organizations, which currently do not exist, is becoming necessary. Another important limitation is the cost of developing and training AI and the need to have a lot of quality data to achieve reliable results. Data should be standardized as much as possible, considering the variability of clinical conditions, to avoid generating unpredictable algorithms. Finally, randomized controlled clinical trials are needed to evaluate the appropriateness of AI algorithms.

In general, the scientific community remains confident about future applications of AI in surgery, screening, and diagnosis. As discussed in our review, deep learning and machine learning systems are expected to improve the recognition and classification of potentially malignant and malignant lesions. Furthermore, AI models are showing the potential to enable future diagnostic improvement and management of patient clinical data.

In our opinion, academic societies and surgical training programs should promote a basic understanding of clinical AI. At the same time, AI should be further developed and improved as an aid in the process of surgical decision-making. Ultimately, AI in surgery should earn the trust of surgeons by demonstrating performance benefits in terms of patient care.

## 5. Conclusions

The aim of this systematic review is to describe the state of the art and potential future developments of AI as an aid in the screening and diagnosis of main surgical diseases. The review of literature indicates that the application of AI has already shown promising results in improving the efficiency and accuracy of diagnosis and treatment in several types of cancer, such as breast and colorectal. In other fields, such as thyroid pathology, AI is still facing several problems: unreliability in cytopathological diagnosis, difficulty in discriminating follicular lesions, and inaccurate prognosis. The future prospect is to speed up, implement, and improve the automaticity with which AI recognizes, differentiates, and classifies pathological lesions. However, RCTs should be performed to evaluate long-term functional outcomes.

## Use of AI tools declaration

The authors declare they have not used Artificial Intelligence (AI) tools in the creation of this article.
